# *Clostridium difficile* isolated from faecal samples in patients with ulcerative colitis

**DOI:** 10.1186/s12879-019-3965-8

**Published:** 2019-04-30

**Authors:** Parisa Shoaei, Hasan Shojaei, Mohammad Jalali, Farzin Khorvash, Sayed Mohsen Hosseini, Behrooz Ataei, Bahareh Vakili, Fatemeh Ebrahimi, Hossein Tavakoli, Zahra Esfandiari, J. Scott Weese

**Affiliations:** 1 Nosocomial Infection Research Center, Isfahan University of Medical Sciences, Isfahan, Iran; 2 Department of Microbiology, School of Medicine, Isfahan University of Medical Sciences, Isfahan, Iran; 3 School of Food Science and Nutrition, Isfahan University of Medical Sciences, Isfahan, Iran; 4 Epidemiology and biostatics department, Isfahan University of Medical sciences, Isfahan, Iran; 5 Infectious Diseases and Tropical Medicine Research Center, Isfahan University of Medical Sciences, Isfahan, Iran; 6 Department of Microbiology, Science and Research Branch, Islamic Azad University, Tehran, Iran; 7 Department of Microbiology, Islamic Azad University of Falavarjan, Isfahan, Iran; 8 Department of Physiology and Pathophysiology, University of Manitoba, Winnipeg, MB Canada; 9 Department of Research and Development, Vice Chancellory for food and drug, Isfahan, Iran; 10 Department of Pathobiology and Centre for Public Health and Zoonoses, Ontario Veterinary College, University of Guelph, Guelph, Canada

**Keywords:** *Clostridium difficile*, Ulcerative Colitis, IBD patients, PCR-Ribotyping, Multilocus sequence typing

## Abstract

**Background:**

Ulcerative colitis (UC) is an inflammatory bowel disease (IBD) that is widely identified worldwide. This study aimed to investigate the phenotypic characterization and molecular typing of *Clostridium difficile* isolates among patients with UC at an inflammatory bowel disease clinic in Iran.

**Methods:**

In this cross-sectional study, conducted from April 2015 to December 2015, 85 UC patients were assessed for *C.difficile* infection (CDI). *C. difficile* isolates were characterized based on their toxin profile and antimicrobial resistance pattern. Multi-locus sequence typing analysis (MLST) and PCR ribotyping were performed to define the genetic relationships between different lineages of toxigenic strains.

**Results:**

The prevalence of *C. difficile* isolates was 31.8% (27/85) in patients, of those 15 patients (17.6%) had CDI. Three different sequence types (STs) identified based on MLST among the toxigenic isolates, that is ST54 (33.3%), ST2 (53.3%), and ST37 (13.6%).

*C. difficile* strains were divided into four different PCR-ribotypes (012, 014, 017 and IR1). The most common ribotype was 014 accounting for 48.3% (7/15) of all strains. The strains isolated during the first episode and recurrence of CDI usually belonged to PCR ribotype 014 (ST2). A high rate of CDI recurrence (14.1%, 12/85) experienced in UC patients. Colonization of the gastrointestinal tract with non-toxigenic *C. difficile* strains was frequent among patients with mild disease.

All *C. difficile* isolates were susceptible to metronidazole, and vancomycin, 86 and 67% of isolates were resistant to clindamycin and erythromycin respectively. There was no correlation between the toxin type and antibiotic resistance (*p* > 0.05).

**Conclusion:**

Overall CDI is rather prevalent in UC patients. All patients with CDI experienced moderate to severe disease and exposed to different antimicrobial and anti-inflammatory agents. Close monitoring and appropriate management including early detection and fast treatment of CDI will improve UC outcomes.

**Electronic supplementary material:**

The online version of this article (10.1186/s12879-019-3965-8) contains supplementary material, which is available to authorized users.

## Background

*Clostridium difficile*, a gram-positive anaerobic bacterium, is the leading cause of pseudomembranous colitis, nosocomial and antimicrobial-associated diarrhea [1, 2]. Increases in morbidity, mortality and relapse rates, along with the emergence of community-associated disease, have heightened concern about CDI internationally. The pathophysiology of *C. difficile* involves colonization of the intestinal tract and toxin production. Toxin A encoded by *tcdA*, and toxin B encoded by *tcdB*, are the most important recognized virulence factors, while another toxin, CDT (encoded by *cdtA/cdtB*) is present in a subset of toxigenic strains [3, 4].

Various molecular typing methods have been applied to characterize outbreaks and describe endemic CDI and *C. difficile* colonization. Hypervirulent clones such as ribotype 027 and to a lesser degree ribotype 078 have been involved in severe nosocomial outbreaks of CDI [3, 4]. Multilocus sequence typing analysis (MLST) for *C. difficile* has also been developed to study clonal relations of the bacterial populations [5, 6]. Recent MLST studies on *C. difficile* have focused on human isolates, animal and food strains [6, 7].

Ulcerative colitis (UC) is an inflammatory bowel disease (IBD) that is widely identified worldwide. The clinical outcome of UC is highly variable from mild to an aggressive disease that may require a colectomy [8]. Determining risk factors that influence disease course is an important clinical issue. Many studies have shown that UC patients have a high risk of *Clostridium difficile* infection (CDI) when compared with healthy population or individuals with Crohn’s disease [9, 10]. CDI can worsen the prognosis of recently diagnosed patients with UC, increasing the risk of colectomy, postoperative complications, and death [8, 11]. There is a report indicating that the incidence of CDI in IBD patients in a nationwide data analysis doubled from 2.66 to 5.12% over a 7 years period in the USA [12, 13]. Potential risk factors for acquiring CDI in IBD patients are similar to those in the non-UC population and include the use of broad-spectrum antimicrobial drugs, especially fluoroquinolones, age over 65, chemotherapy and hospitalization [13–16]. In addition, decreased intestinal microbial diversity along with an inadequate immune response may play a causative role in the development CDI. Moreover, diagnosis and appropriate management of CDI in the setting of IBD is difficult due to overlap symptomatology, as both infection and disease flare present with similar symptoms of elevated inflammatory biomarkers and diarrhea [17]. There is a strong recommendation that all patients with IBD, hospitalized with disease flare and patients who develop diarrhea in the setting of quiescent disease must undergo testing for CDI [18].

Data on the prevalence of CDI in UC patients have come from developed countries. Since limited information is available on the occurrence of CDI in UC patients in Iran or other Middle Eastern countries, we carried out this study to investigate the prevalence of CDI in patients with UC and, to characterize *C.difficile* isolates in UC patients.

## Methods

This was a cross-sectional study conducted on ulcerative colitis patients referred to the Isfahan University of Medical Sciences inflammatory bowel disease clinics in Isfahan, Iran between April 2015 and December 2015. Patients between 18 and 65 years old with a documented diagnosis of ulcerative colitis were included. Patients with chronic obstructive pulmonary disease, severe liver dysfunction, end-stage renal disease, malignancy, and immunodeficiency syndromes were excluded. The demographic information and potential risk factors of patients such as age, gender, previous surgery, all drugs used by patients and antibiotic treatment within 8 weeks before the time of *C. difficile* detection were recorded. Diagnosis of UC was based on clinical signs and symptoms combined with disease activity, histologic, endoscopic, and radiological results according to the Porto criteria and Truelove-Witts activity index [19]. The moderate or severe disease was defined as symptomatic UC (fever, blood in the stool, number of stool specimen, hemoglobin level and elevation of Erythrocyte sedimentation rate (> 30) and with a Truelove–Witts score greater than 4 points) [20–22].

*Clostridium difficile* infection most commonly defines as the presence of *C. difficile* toxin in the context of characteristic clinical manifestations including diarrhea and abdominal pain in contrast with *C. difficile* colonization in healthy individuals. Because of UC and CDI symptoms overlap we described CDI as three or more daily bowel movements for a period of at least 48 h in the setting of previously quiescent UC disease [23].

CDI recurrence was described based on looser bowel movement numbers or developed new signs of severe colitis that last for more than two days [13].

A total of 170 fecal samples were collected from 85 UC (2 samples per patient) ranging in age from 20 to 65 years. The first set of samples were collected on May 2015 and the second set was taken about 2–3 months later. Stool samples collected in sterile collectors and immediately transferred into the laboratory of Infectious Diseases and Tropical Medicine Research Centre, Isfahan, Iran and preserved at − 70 °C during the analysis period. Patients with UC were treated with anti-inflammatory and steroids drugs in accordance with clinical protocols. Patients with CDI (*tcdA* and or *tcdB* positive isolates) treated with metronidazole for 2 weeks. Stools specimens were screened for the presence *C. difficile*. Stools specimens were analyzed for the presence of other enteropathogenic organisms (*E. coli*, *Salmonella spp*, *Shigella spp*, and *Campylobacter jejuni*).

### *Clostridium difficile* culture

Selective, enrichment culture was performed [20]. Briefly, about 2 g of stool was inoculated into 10 ml of *C. difficile* moxalactam norfloxacin (CDMN) broth culture. The cultures were incubated in an anaerobic jar in an atmosphere composed of 86% N_2_, 7% H_2,_ and 7% CO_2_ at 37 °C for 48 h by using an Anoxomat system (MART Microbiology B.V., Drachten, Netherlands). One mL of enriched broth was mixed thoroughly with an equal volume of 95% alcohol and held at room temperature for 30 min. The tubes were centrifugated at 2500 g for 5 min and the alcohol supernatant was decanted. The pellets were inoculated by a swab onto the *C. difficile* moxalactam norfloxacin agar (CDMN) and incubated anaerobically for 48 h at 37C°. Negative cultures remained in the incubator for up to 7 days. The colonies characterized with 2–3 mm in diameter, *p*-cresol odor, ultraviolet fluorescence (365 nm), typical Gram stain morphology, positive malachite green for spore and positive biochemical reactions such as L-proline aminopeptidase test (Prodisk, Remeb, Lenexa, KS, USA) were identified as *C. difficile* and stored at 4 °C [4, 24].

### Molecular identification of *C. difficile*

DNA extraction was performed using the modified Pitcher et al., procedure (1989). Briefly, Cultures of *C. difficile* strains grown in BHI broth were centrifuged and cells were treated with lysozyme (50 mg/ml) and resuspended in TE (Tris, 10 mM; EDTA, 50 mM; pH 8.0). Guanidium thiocyanate and sarkosyl were added to the mixture for protein denaturation [25, 26]. All isolates were screened for the presence of the genes encoding toxin A and B (*tcdA* and *tcdB*), binary toxins (*cdtA, cdtB*) and triose phosphate isomerase (*tpi*) [24, 27]. Multiplex PCR amplification performed in a thermocycler (Eppendorf, Germany). The 25 μl reaction mixture included 1× PCR buffer, 250 μM of each dNTPs, 10 pM of primers (*tcdA, tcd B*), 5 pM of primers (*tpi*), 1 U Taq polymerase (Cinna Gene, Iran) and 100 ng of DNA. Amplification was carried out in a touchdown protocol [4, 24, 27]. *C. difficile* ribotype 027 was used as positive control for molecular and microbiological analysis. *C. perfringens* 450 MTCC (Microbial Type Culture Collection) served as the negative control [28].

### Disk diffusion antimicrobial susceptibility testing

The following antimicrobial susceptibility disks were used for antimicrobial susceptibility test metronidazole (5 μg), vancomycin (30 μg), clindamycin (2 μg), moxifloxacin (5 μg), fusidic acid (10 μg), erythromycin (15 μg), rifampicin (5 μg), (Rosco Diagnostica A/S NEO-SENSITABS TM, Denmark). All tests were performed on Brucella Blood Agar containing vitamin K1 (1μg/mL), haemin (5 μg/mL) and 5% defibrinated sheep red blood cells [29]. The zone diameters were read at 100% inhibition. For the preparation of inoculum, inoculation, and incubation we followed the 15–15-15-min rule as recommended by the European Committee on Antimicrobial Susceptibility Testing (EUCAST) (http://www.eucast.org). Strains were deemed susceptible or resistant to the test antibiotic according to documented pharmacological breakpoint values [30]. The antimicrobial agents tested were selected because of the emergence of reduced susceptibility.

### Multilocus sequence typing (MLST)

Multilocus sequence typing (MLST) with seven housekeeping genes (*adk, atpA, dxr, glyA, recA, sodA, and tpi*) was performed as described by Griffiths et al., 2010 [5]. The amplified products were sequenced by Bioneer Corporation in South Korea. The DNA sequences of the 7 genes were submitted to the MLST database to determine the sequence type (ST).

### PCR-Ribotyping analysis

Isolates were subjected to PCR-ribotyping as described by Bidet et al. [12]. Interpretation of ribotyping results was performed by visual identification. Ribotype patterns were designated by internal nomenclature. Reference strains of ribotype 027 and 078 were available for comparison.

### Statistical methods

Data were presented as count and percentage. A first univariate logistic regression model was fitted on each independent variable, and a multivariate regression model with adjustment for the effects of other covariates was used. Variables that were significant in univariate models (*p* < 0.05) were entered into the multivariate model. Selection of variables in the multivariate model was based on a stepwise procedure. We estimated Odds Ratios (ORs) and 95% confidence intervals for each of clinical factors using logistic regression models. All probabilities were two-tailed and a *p*-value of < 0.05 defined statistically significant. Statistical analysis was performed using the statistical software SPSS, version 16.

## Results

Out of 85studied patients, 27 *C. difficile* isolates (31.8%) were recovered from their stool specimens, including 15 (17.6%) with CDI (toxigenic isolates carried one or both *tcdA* and *tcdB* genes) and 70 (82.4%) with non-CDI. The toxigenic isolates detected in both stools samples of patients with CDI. None of the specimens were positive for toxin A alone or binary toxin. *Salmonella spp*, *Shigella spp*, *E. coli,* and *Campylobacter jejuni* were not detected. The use of antibiotic was identified in 74 (87%) patients in the 8 weeks prior to CDI diagnosis. (Table 1).

**Table 1:** Clinical characterizations of 85 Ulcerative Colitis patients in CDI and Non CDI groups

Variables		CDI patients (15)	Non-CDI patients (70)		Univariate analysis
		Toxigenic *C.difficile* strains, (A + B+ or A-B+), *n* = 15	Negative *C.difficile* strains, *n* = 58	Non toxigenic strains, *n* = 12	*P* value
		Count (%)	Count (%)	Count (%)	
Male		8 (53.3)	37 (63.8)	6 (50)	0. 08
Severity of UC					
Mild		0	38 (65.5)	11 (91.7)	
Moderate to severe		15 (100.0)	20 (34.5)	1 (8.3)	0.001
Previous surgery		10 (66.7)	16 (27.6)	6 (50)	0.02
Antibiotic treatment within 8 weeks prior to CDI (metronidazole, Cyclosporine, Clindamycin, cephalosporin, …)	One	4 (26.7)	18 (31.0)	3 (25.0)	0.2
	Two	6 (40.0)	18 (31.0)	4 (33.3)	
	Three	5 (33.3)	12 (20.7)	4 (33.3)	
Steroids		15 (100)	20 (34.5)	4 (33.3)	0.001
Anti-inflammatory drugs	Mesalamine	8 (53.3)	21 (36.2)	4 (33.3)	0.01
	Sulfasalazine	11 (73.3)	22 (37.9)	4 (41.7)	
History of colectomy		5 (33.3)	11(18.9)	3 (25.0)	0.03
Age at diagnosis (years), mean ± SD		46.5 ± 11.4	42.3 ± 13.1	41.2 ± 14.3	0.07

Legends: *UC* ulcerative colitis, Mesalamine: 5-aminosalicylic acid (5-ASA), *SD* standard deviation

Toxigenic *C. difficile* strains were divided into four different PCR-ribotypes (012, 014, 017 and IR1) patterns. The most common ribotype was 014 accounting for 48.3% (7/15) of all toxigenic *C.difficile* isolates followed by the ribotypes 012 (26.7%, 4/15) and 017 (13.6%, 2/15) respectively. Three different sequence types (STs) identified based on MLST among the toxigenic isolates, ST54 (*n* = 5, 33.3%, *tcdA*^*+*^*, tcdB*^*+*^*, CDT*^*−*^), ST2 (*n* = 8, 53.3%, *tcdA*^*−*^*, tcdB*^*+*^*, CDT*^*−*^), and ST37 (*n* = 2, 13.6%, *tcdA*^*−*^*, tcdB*^*+*^*, CDT*^*−*^). Strains isolated during the first episode and recurrence of CDI belonged to PCR ribotype 014 (ST2) (Additional file 1). A high rate of CDI recurrence (14.1%, 12/85) experienced in UC patients. Clinical recurrences of CDI were diagnosed within one month of the antibiotic treatment. Among 85 patients, PCR ribotyping and MLST analysis showed *C.difficile* re-infection throughout the incident of UC. The most frequent isolates belonged to ribotype 014 (ST2). Two out of 12 patients (17%) of studied patients were recognized re-infection by an identical strain of *C. difficile* (ribotype 014/ST2). Colonization of the gastrointestinal tract with non-toxigenic *C. difficile* strains (ribotype IR5, ST15, *tcdA*^*−*^*, tcdB*^*−*^*,* CDT^*−*^) was frequent (86.7%, 13/15) among patients with mild disease.

All *C. difficile* strains were susceptible to metronidazole (range 23–45 mm) and vancomycin (range 19–28 mm). Only one isolate from a patient aged 60 with severe disease was resistant to fusidic acid, while the remaining isolates (96.3%, 26/27) were susceptible to it (range 19–28 mm). Twenty four out of 27 patients (89%) were susceptible to moxifloxacin (range 6–28 mm) and rifampin (range 23–45 mm) while, 23 out of 27 isolates (86%) were resistant to clindamycin, 18 out of 27 isolates (67%) to erythromycin (median range 6–18 mm). There was no correlation between the toxin type and antibiotic resistance (*p* > 0.05).

The stepwise multivariate logistic regression model revealed that patients who used steroids 8 weeks prior to testing for *C. difficile* were more than 6 times more likely to develop CDI than those who didn’t (OR 6.03; 95% CI, 4.6–38.5; *p* = 0.004).

The study findings also revealed that patients with a history of previous surgery, anti-inflammatory treatment, and complicated UC were more likely to develop CDI (Table 2). We did not find a significant association between age, gender, colectomy, recent hospitalization and CDI in UC patients.

**Table 2:** Multivariate logistic regression analysis for occurrence of *C.difficile* infection on UC patients

Risk factors	OR (95% CI)	*P* value
Steroid treatment	6.03 (4.6–38.5)	0.004
Previous surgery	2.5 (1.1–5.8)	0.049
Severity of UC	2.9 (1.9–23.8)	0.011
Anti-inflammatory drugs	1.54 (1.3–5.97)	0.039

Legends; *OR* Odds ratio, *CI* Confidence interval

## Discussion

Toxin-producing *C. difficile* strains are associated with worsening disease in UC patients. Recent decade studies have been shown a steady increase in CDI prevalence especially in UC patients [21, 23, 31]. The prevalence of CDI in UC patient in the present study was estimated as being 17.6%. There are no systematic data to evaluate any increase/decrease of CDI prevalence in general and particularly in UC patients in Iran. However, the existing data from other countries indicate a significantly lower incidence of CDI (2.8–11.1%) in adult UC patients compared to our findings [17, 32]. Recent studies from Europe, Canada, and the United States suggesting a rate of 20–27% community-acquired CDI in the general population [33]. Other recent studies have reported that in the majority of IBD patients, CDI was contracted outside of the hospital and 47.2% of patients acquired CDI from the community [34]. Several factors may explain this higher rate including, patient population and sampling period. Fecal sampling for toxin detection was performed during a follow-up visit, which is regularly settled especially in the presence of worsening and we could detect more CDIs.

We found a significant relationship between the presence of CDI and steroid treatment, previous surgery, the severity of UC and anti-inflammatory treatment (Table 2). Previous studies have demonstrated that in UC patients, CDI is prevalent and colonic complicity, female, recent surgery, colectomy, younger age, and systemic steroid therapy was independently associated with CDI [8, 17, 23, 33–35]. Fourteen percent of our patients showed one or two episodes of recurrences during the study period. Recurrence of CDI among UC patients is a fundamental problem and identification of CDI in early diagnosed UC may be beneficial because superimposed the management of CDI lead to clinical remission of UC [34]. Previous studies showed about 18 to 25% of IBD patients had experienced CDI recurrence within 30 days following treatment with metronidazole or vancomycin [31, 36]. Recurrences of *C. difficile* may be described either by the endogenous persistence of a *C. difficile* strain (relapse) or by contamination of a new strain from the environment (re-infection) [37]. About 17% of studied patients in this study were recognized re-infection by an identical strain of *C. difficile* (ribotype 014/ST2). In other similar studies that performed in hospitalized patients, the rate of re-infection with identical strains was between 38 and 56% [8, 21, 37].

**Fig. 1:**
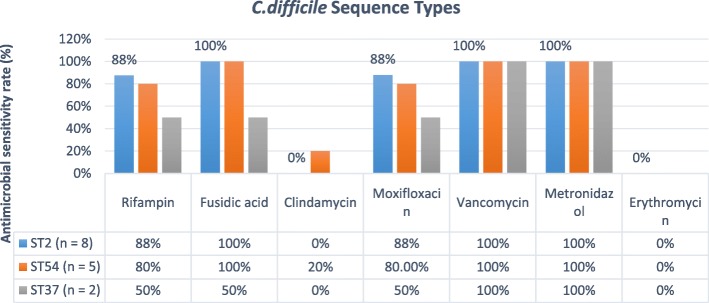
Antimicrobial sensitivity rates among different STs of toxigenic *C. difficile* isolates. Legends: ST, sequence type

All identified *C. difficile* strains in our study showed susceptibility to vancomycin and metronidazole. Recurrence CDI has been observed in patients that had taken metronidazole. Other similar studies showed that patients with an IBD flare and concurrent CDI treated with vancomycin had successful treatment and vancomycin is the first-choice therapy for moderate to severe CDI [17, 23]. Most studies have exhibited that certain antibiotics such as clindamycin, moxifloxacin, and fluoroquinolones carry a higher risk for CDI. Low susceptibility to these antimicrobial agents has been reported in other studies that can be attributed to different antibiotic regimens used [38, 39]. There was no significant difference in the resistance rates between CDI and non-CDI patients with respect to their susceptibility to these antibiotics (Fig. 1). Hypervirulent Ribotype 027 (ST1) also was not found in the present study, nor was ribotype 078 (ST11), based on inference of the lack of *cdtA*/*cdtB* genes in any isolate and MLST analysis. We have previously reported ribotype 078 as a common strain in both humans and meat in Iran so the absence of this strain in the current study was surprising. [4, 40]. Most of CDI in the current study were found to be due to A^−^B^+^ strains (ST2, ST37). Recent studies have reported an increasing number of infections due to A^−^B^+^ strains especially ST37 in Asia although such strains do not produce a binary toxin [41].

In the previous report from Southern India, nontoxigenic *C. difficile* strains identified in a significantly high rate of 90% of UC patients and about 55% of healthy subjects [21]. We found an overgrowth of nontoxigenic *C. difficile* intestinal carriage of the much lower rate in 12 patients (14.2%) of studied patients. Its relatedness to disease pathogenesis and severity in a contaminated environment justifies further investigations.

There are several limitations to our study including small sample size, which have led to underestimating the true prevalence and diversity of circulating *C. difficile* strains and lack of a healthy control group to be compared with the UC patients. This is the first study provides information about different aspects of molecular epidemiology, clinical characteristics, and antibiotic resistance profiles of circulating *C. difficile* strains among Iranian patients with ulcerative colitis. Further research and clinical studies with a larger population should be performed to evaluate the epidemiology of *C. difficile* in this high-risk group.

## Conclusion

Overall, CDI is rather prevalent in UC. All patients with CDI experienced moderate to severe disease and exposed to different antimicrobial and anti-inflammatory agents. Close monitoring and appropriate management including early detection and fast treatment of CDI will improve UC outcomes.

## Additional file

Additional file 1: Normalized dendrogram of the detected isolates of *C.difficile,* PCR- ribotyping fingerprints with the primers 16S–23S. Similarity coefficients are included in the top bar; Dendrogram is color-coded according to sequence types (STs) and toxin types. The similarity was calculated using the Dice coefficient and UPGMA clustering. (DOCX 951 kb)

## Abbreviations

UC: Ulcerative colitis
IBD: Inflammatory bowel disease
CDI: *Clostridium difficile* infection
MLST: Multilocus sequence typing analysis
CDMN: *C. difficile* moxalactam norfloxacin
ST: Sequence type
