# Supplementary material for: Clostridium difficile isolated from faecal samples in patients with ulcerative colitis
Source: BMC Infect Dis. 2019 Apr 30;19:361. doi: 10.1186/s12879-019-3965-8 (PMC6492486; doi:10.1186/s12879-019-3965-8)
Supplement: Supplementary file 1 — Normalized dendrogram of the detected isolates of C.difficile, PCR- ribotyping fingerprints with the primers 16S–23S. Similarity coefficients are included in the top bar; Dendrogram is color-coded according to sequence types (STs) and toxin types. The similarity was calculated using the Dice coefficient and UPGMA clustering. (DOCX 951 kb) [file 12879_2019_3965_MOESM1_ESM.docx]

Additional File 1

Figure S1.a: *C. difficile* culture on CDMN agar, b:

Figure S2.Characterization of the 3 main *C.difficile* toxigenic types by multiplex PCR, *tpi* fragment 230 bp, non deleted tcdA fragment (369 bp), *tcdB* fragment.


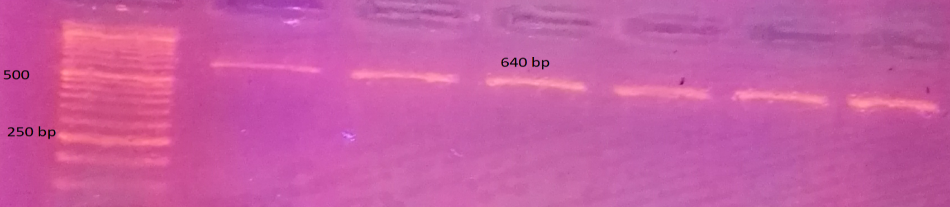


Figure S3. PCR- electrophoresis of *tpi* gene (housekeeping gen)


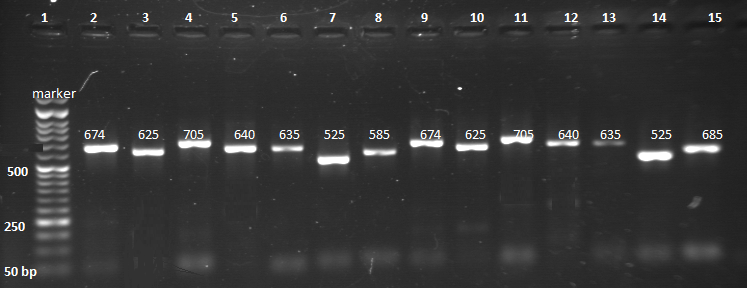


Figure S4: Characterization of 7 houskeeping genes for MLST analysis by PCR, *atpA* fragment 674 bp, *glyA* fragment 625 bp, *recA* fragment 705 bp, *tpi* fragment 640 bp, *adk* fragment 635 bp, *sodA* fragment 585 bp.


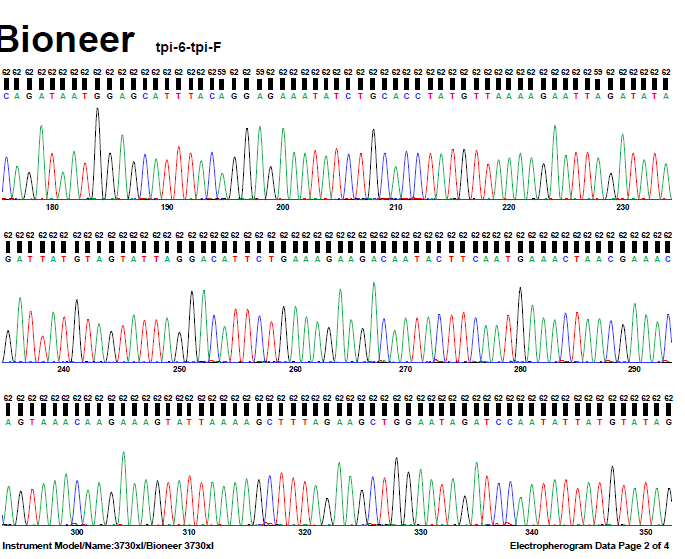


Figure S5. A part of PCR sequencing *tpi* gene.


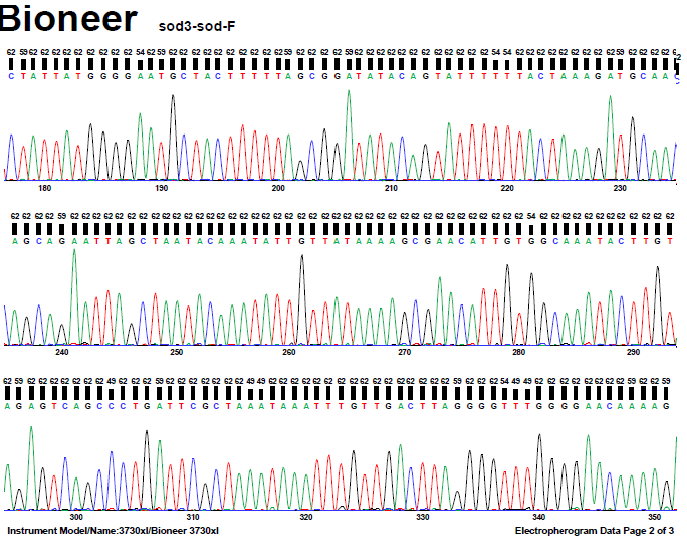


Figure S6. A part of PCR sequencing of *sodA* gene.


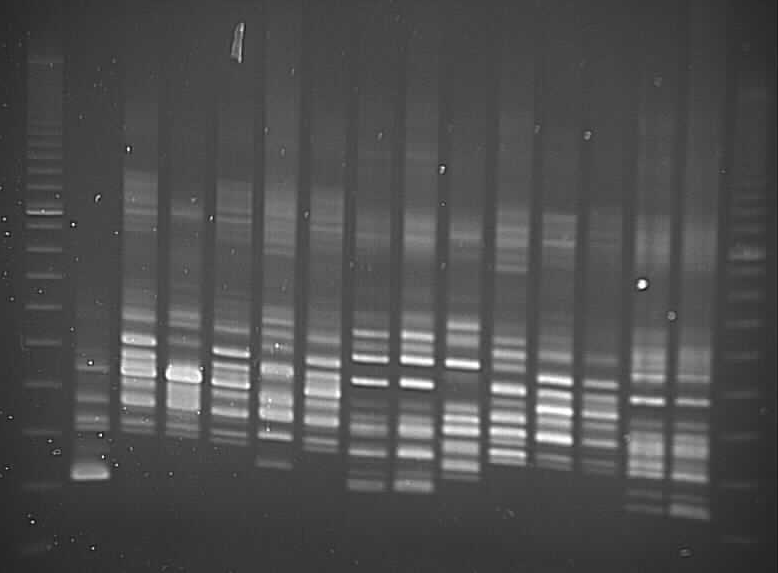


Figure S7. PCR- Ribotyping of *C.difficile* toxigenic isolates.
